# The impact of healthy nutrition education based on traffic light labels on food selection, preference, and consumption in patients with acute coronary syndrome: a randomized clinical trial

**DOI:** 10.1186/s12889-024-18805-2

**Published:** 2024-05-17

**Authors:** Fereshteh Sadeghi, Shahzad Pashaeypoor, Akbar Nikpajouh, Reza Negarandeh

**Affiliations:** 1 Department of Community Health and Geriatric Nursing, School of Nursing and Midwifery, Tehran University of Medical Sciences, Tehran, Iran; 2 Rajaie Cardiovascular Medical and Research Center, Iran University of Medical Sciences, Tehran, Iran; 3 Nursing and Midwifery Care Research Center, School of Nursing and Midwifery, Tehran University of Medical Sciences, Tehran, Iran

**Keywords:** Traffic light labels, Acute coronary syndrome, Healthy eating style, Healthy food selection, Food preference, Food consumption

## Abstract

**Background:**

Acute Coronary Syndrome is the most common heart disease and the most significant cause of death and disability-adjusted life years worldwide. Teaching a healthy eating style is one preventive measure to prevent the disease’s recurrence. This study aimed to determine the effect of healthy nutrition education with the help of traffic light labels on food selection, preference, and consumption in patients with acute coronary syndrome.

**Methods:**

This randomized, single-blinded clinical trial was conducted with 139 participants (66 in the intervention group and 73 in the control group) from January 2021 to August 2021 in Shaheed Rajaie Hospital, Tehran, Iran. The control group received standard training. The intervention group, besides this, received additional bedside training with an educational poster on traffic light labels from the research team during their final hospitalization days. Data were collected using a researcher-made questionnaire on food selection, preference, and consumption.

**Results:**

The Brunner-Munzel test showed no significant difference between the two groups in terms of selection (*P* = 0.127), preference (*P* = 0.852), and food consumption (*P* = 0.846) in the baseline, while after the intervention, there were significant differences in selection (*P* > 0.001), preference (*P* > 0.001), and consumption (*p* < 0.004). Comparing the difference between the two groups in the difference between the before and after scores for selection (*p* < 0.001), preference (*p* < 0.001), and food consumption (*p* = 0.011) with the Brunner-Munzel test indicated a significant difference in all outcome variables.

**Conclusions:**

Teaching healthy eating styles with the help of traffic light labels affected food selection, preference, and consumption and led to healthier diets in these patients.

**Clinical trial registration number:**

Clinical trial registration: It was prospectively registered in the Iran Clinical Trials Registration Center on this date 30/10/2020 (IRCT20200927048857N1).

**Supplementary Information:**

The online version contains supplementary material available at 10.1186/s12889-024-18805-2.

## Background

Acute coronary syndrome (ACS) is the most common heart disease and the most significant cause of death and disability-adjusted life years (DALYs) worldwide [1]. ACS is a group of clinical symptoms corresponding to acute myocardial ischemia and has a significant clinical and financial impact. Clinical variants of ACS include unstable angina and acute myocardial infarction (AMI) with or without ST-segment elevation [2]. Globally coronary artery diseases cause approximately 7 million yearly deaths [3]. Coronary artery diseases are also the first cause of death in people over 35 years of age in Iran and cause 39.3% of all deaths in the country [4].

These patients are discharged from the hospital after treatment, but due to the chronic nature of the disease, people who survive the first ischemic attack are at greater risk for cardiovascular events in the future [5]; ACS patients are 20% more at risk than people without coronary artery disease during the five years after an ischemic attack [6, 7]. The most important behavioral risk factors for cardiovascular diseases are inactivity, an unhealthy diet, and smoking. By changing these risk factors, many cases of cardiovascular diseases can be prevented [8]. Secondary prevention guidelines encourage these patients to consume heart-protecting foods (fruits, vegetables, olive oil, and whole grains) and avoid consuming heart-damaging foods such as sweet drinks, processed meats, and foods containing trans fatty acids. It is also encouraged to limit the amount of sodium consumption.

Despite the availability of these guiding principles, previous studies have reported poor patient adherence to this advice, as patients, especially in low-income countries, tend to maintain their pre-heart attack diet [9].

The extraordinary increase in food-related diseases is due to poor eating habits [10]. Unfortunately, even consumers who are motivated to choose healthy foods sometimes fail to accurately assess the healthiness of food due to barriers to understanding nutrition information and utilizing it [11].

Nutritional information is provided through nutritional education [12]. Nutritional education programs can increase nutritional knowledge and improve nutritional behaviors and therefore help prevent many chronic diseases such as cancer, diabetes and cardiovascular diseases [13]. The findings of Pem et al.‘s study showed that a nutrition education program is a promising strategy for nutritional behaviors. Participants who received nutrition education had fundamental changes in their behavior, knowledge, and attitude towards healthier eating, the amount of fruit consumption increased while the consumption of snacks rich in sugar and fat decreased. However, the consumption of vegetables, energy intake, body mass index and physical activity level did not change after training [14]. In Mohammadi et al.‘s study, the educational method was effective in improving the level of nutritional awareness, weight loss motivation, and people’s performance, but people’s attitudes towards the correct consumption of food did not change [15].

The traffic light label (TLL) is a type of front-of-package food labeling (FOPL) proposed by the British Food Standards Agency in 2006. The TLL is an effective tool for conveying complex nutritional information that, by being placed on the front of food packaging, can potentially contribute to reducing the consumption of products with high levels of fat, salt, and sugar. A feature of this label is that it categorizes according to color, with “little” recognized by green, “medium” by yellow, and “high” by red. The amount of energy is also shown in white [10]. In Iran, since 2014, the Food and Drug Organization has required TLL with five factors (energy, fat, salt, sugar, and trans fat) on all packaged food products, except for some exceptions. The energy is shown in kilocalories; the other factors are in grams per 100 g or 100 ml of the food. By providing nutritional information on the front of food packaging, TLL helps people choose food according to their physical conditions and personal needs and thus reduces the burden of chronic diseases [16]. This label can be understood without having nutritional literacy, so it has the potential to be used by the majority of people in society [17]. In addition, the need to disclose nutritional information can encourage food manufacturers to improve the nutritional profile of their products [18].

Previous evidence has shown that food labeling in a cafeteria in Boston, as well as in a sports recreation venue in Canada and a restaurant in Taiwan, increased the purchase of healthier products and decreased the purchase of unhealthy products [19–21]. In contrast, a study in England and Australia reported no change in the sale of healthier products after introducing the TLL label [22, 23]. Despite the limited studies investigating the effect of TLL on the selection and consumption of food items in healthy populations, no study was found that used TLL to educate patients with a history of hospitalization. Therefore, we hypothesized that introducing TLL to ACS patients would improve their food choices, preferences, and consumption, enabling them to prevent chronic diseases. This study was conducted to determine the effectiveness of using TLL in patient education on food selection, preference, and consumption in ACS patients.

## Methods

### Study design

In this randomized, single-blind clinical trial, from January 2021 to August 2021, we enrolled 170 patients with ACS and who met the eligibility criteria. They had been referred to Shaheed Rajaie Cardiovascular Medical & Research Center, Tehran, Iran.

### Study population

The inclusion criteria were: ACS diagnosis confirmed by a cardiologist, age 20 to 60 years, first admission due to ACS attack in Shaheed Rajaie Hospital, absence of communication and cognitive problems (with self-report and patient file contents), lack of vision problems or color blindness, having the ability to understand the Persian language, being able to read and write, and not having a history of participating in educational classes on the same subject. Exclusion criteria were: having special dietary restrictions, being hospitalized again during the study, death, or unwillingness to be in the study.

### Intervention

During the course of their hospitalization, all participants were provided with standard training. In addition to this, the intervention group received supplementary training on Traffic Light Labelling (TLL) from the research team, facilitated through an educational poster. This additional training was administered bedside in the concluding days of their hospital stay. Each training session lasted 15 to 20 min. The training encompassed an overview of the TLL system and a detailed explanation of types of fat. An exemplar packaged food product, labeled with green (indicating salt), red (indicating sugar and fat), and yellow (indicating trans fatty acid), was utilized to illustrate the TLL system.

### Randomization

After taking the informed consent and conducting the pre-test, the participants were randomly assigned to two groups: routine care (control) and TLL food label training (intervention).

### Measuring study outcomes

#### Demographic information

Demographic information includes age, gender, marital status, place of residence, body mass index, level of education, occupation, type of insurance, household income, smoking, family history of heart attack, family history of angina pectoris, history of diabetes, a history of high blood pressure, and a history of high cholesterol.

#### Food preference

The questionnaire pertaining to food preferences comprised four dichotomous questions, wherein participants were presented with a choice between healthy and unhealthy options. The options included a preference for low-salt (healthy) versus salty (unhealthy) cheese, high-fat (unhealthy) versus low-fat (healthy) milk, sugar-containing (unhealthy) versus sugar-free (healthy) soft drinks, and solid (unhealthy) versus liquid (healthy) vegetable oil. A scoring system was implemented where the selection of an unhealthy option was awarded one point, while the choice of a healthy option garnered two points.

#### Food selection

The food selection questionnaire comprised ten items. The respondents indicated their choice on a Likert scale ranging from 1 (never) to 5 (almost always) [5]. Choosing healthier food in this questionnaire means that the participants buy packaged food based on the nutrition label. That is, they read the nutrition label when buying food, specifically in search of information on the amount of sugar, salt, fat and trans fatty acid and other nutritional information. A higher score means healthier food choices.

#### Food consumption

The food consumption questionnaire asked about the frequency of 32 food items harmful to heart health and how much food they ate during the previous month. They received 1 point for eating something six or more times a month and 5 points for never eating it. If the participants used fewer of these items, they would get a higher score, indicating a healthier consumption.

### Validity and reliability of the questionnaires

The food preference, selection, and consumption questionnaires was developed for this study by generating questions through a comprehensive review of existing literature. These questions were deliberately designed to encompass three distinct domains: preference, choice, and consumption of food. (please see supplementary file). To ensure face and content validity, the questions were meticulously reviewed by an expert panel. This panel was composed of faculty members from various disciplines, providing a multidisciplinary perspective on the content. The reliability of the questionnaire was done by the test-retest method for 20 samples. The intra-cluster correlation coefficient was estimated for food selection, food preference, and food consumption questionnaires, and the results were ICC: 0.90 (95% CI: 0.75, 0.96), ICC: 0.99 (95% CI: 0.97, 0. 99), and ICC: 1, respectively. Internal consistency was also estimated using Cronbach’s alpha method, and it was 0.79 for food selection, 0.63 for food preference, and 0.72 for food consumption.

### Sample size and statistical analysis

The sample size for comparing two independent means with an effect size of 0.5 was calculated using G*power. The type I error was 0.05, and the type II error was 0.20. The sample size was 67 per group, but it was increased by 20% to 85 people to allow for dropouts.

Data entry and cleaning were performed using SPSS software. Descriptive statistics and inferential tests were applied to compare the individual and disease characteristics of the two groups. chi-square or Fisher exact tests were used for comparing categorical variables between the intervention and control groups. Next, the assumptions for mixed model ANOVA were checked. The data violated the normality and homogeneity of variance assumptions, so a nonparametric alternative was employed. The Brunner-Munzel test was used to compare the scores of the three outcome variables at baseline and post-intervention, as well as the difference scores between pre-and post-intervention. The Brunner-Munzel test is a nonparametric test of the null hypothesis that when values are taken one by one from each group, the probabilities of getting large values in both groups are equal. The analysis was conducted using JAMOVI software version 2.3.18.0. A significance level of *p* < 0.05 was adopted for all tests. The study was blinded as the statistician did not know the allocation of the patients.

This study was approved by the Organizational Ethics Committee of the Tehran University of Medical Sciences Faculty of Medicine and was registered in the Iranian Clinical Trials Registration Center on this date 30/10/2020 (IRCT20200927048857N1). Before participating in the research, an informed consent form was signed by all participants. It was explained to the patients that refusing to be part of the study would not affect their treatment. Patients were also assured that their identity would remain anonymous and their data would be confidential.

## Results

### Study population

**Fig. 1:**
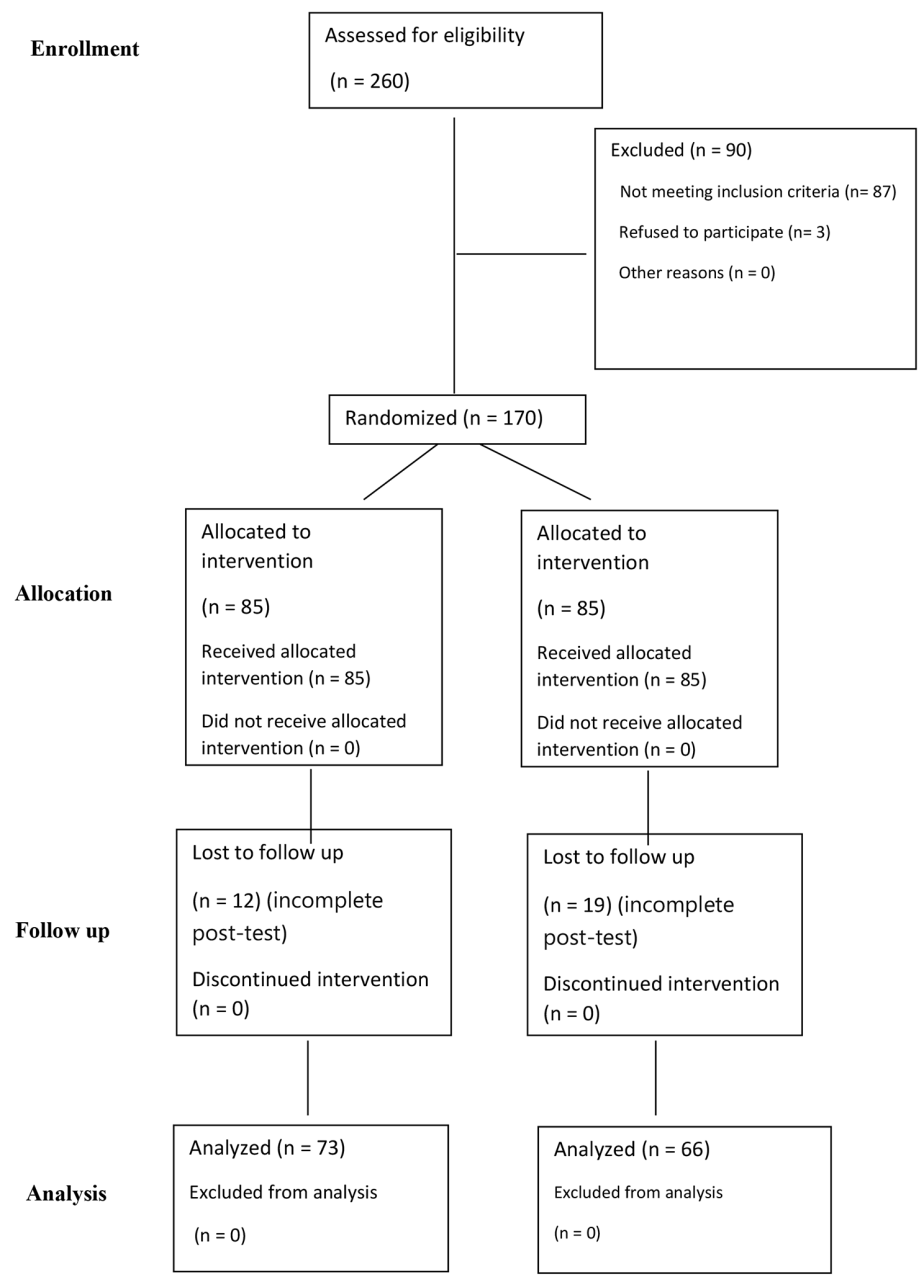
The CONSORT diagram shows the participants’ flow through each stage of a randomized trial

Two hundred and sixty people were checked for eligibility to participate in the study, from January 2021 to August 2021 and 87 were excluded due to not meeting the inclusion criteria. Three people refused to sign informed consent to participate, and 31 people (12 in the control and 19 in the invention groups) did not attend to post-test. Finally, the data of 66 people in the intervention group and 73 in the control group were analyzed (Fig. 1).

Most participants in both groups were aged 50 to 60 (42 in the intervention and 47 in the control). The intervention and control groups were homogenous in demographic and medical history variables (*p* > 0.05) except for diabetes history (*P* = 0.012) (Table 1).

**Table 1:** Demographic characteristics of acute coronary syndrome patients in two intervention and control groups

Variable		Group		Test results
		intervention (66 participants)	Control (73 participants)	
**Age (years)**	20–29 30–39 40–49 50–60	1(1/5) 5(7/6) 18(27/3) 42(63/6)	0 9(12/3) 17(23/3) 47(64/4)	*P* = 0/569^a*^
**gender**	female male	7(10/6) 59(89/4)	10(13/7) 63(86/3)	*P* = 0/578^b*^
**occupation**	Unemployed housewife Retired employed	2 [3] 5(7/6) 7(10/6) 52(78/8)	2(2/7) 8 [11] 10(13/7) 53(72/6)	*P* = 0/867^a*^
**Body mass index**	Normal weight Overweight Grade 1 obesity Grade 2 obesity	17(25/8) 36(54/5) 11(16/7) 2 [3]	19(26) 38(52/1) 13(17/8) 3(4/1)	*P* = 1^a*^
**Marital status**	Single married Divorced	3(4/5) 61(92/4) 2 [3]	3(4/1) 66(90/4) 4(5/5)	*P* = 0/9^a*^
**Place of residence**	City Village	62(93/9) 4(6/1)	71(97/3) 2(2/7)	*P* = 0/423^a*^
**Level of education**	Under diploma Diploma and Associate degree Bachelor Masters degree and higher	25(37/9) 25(37/9) 10(15/2) 6(9/1)	25(34/2) 31(42/5) 12(16/4) 5(6/8)	*P* = 0/905^b*^
**Type of insurance**	Basic Basic and supplementary no insurance	33(50) 24(36/4) 9(13/6)	38(52/1) 28(38/4) 7(9/6)	*P* = 0/756^b*^
**Household income**	Under 2 million tomans 2 to 5 million tomans 5 to 10 million tomans Above 10 million tomans	10(15/2) 33(50) 17(25/8) 6(9/1)	13(17/8) 34(46/6) 17(23/3) 9(12/3)	*P* = 0/884^b*^
**Smoking**	Yes No	37(56/1) 29(43/9)	38(52/1) 35(47/9)	*P* = 0/636^b*^
**Family history of heart attack**	Yes No	27(40/9) 39(59/1)	30(41/1) 43(58/9)	*P* = 0/982^b*^
**Family history of angina pectoris**	Yes No	9(13/6) 57(86/4)	9(12/3) 64(87/7)	*P* = 0/819^b*^
**History of diabetes**	Yes No	7(10/6) 59(89/4)	20(27/4) 53(72/6)	*P* = 0/012^b^
**History of high blood pressure**	Yes No	23(34/8) 43(65/2)	26(35/6) 47(64/4)	*P* = 0/925^b*^
**History of high cholesterol**	Yes No	19(28/8) 47(71/2)	29(39/7) 44(60/3)	*P* = 0/176^b*^

^*a*^ *Fisher’s exact test*

^*b*^ *Chi-square test*

^*^ *Statistically significant (p-Value > 0.05)*

The baseline scores of the three outcome variables did not differ significantly between the two groups, indicating their comparability at the start of the intervention. However, after the intervention, the scores of the three outcome variables showed significant differences between the two groups, favoring the intervention group. To control for the effects of the baseline scores, the difference scores between pre- and post-intervention were calculated and compared using the Brunner-Munzel test. The results revealed a significant difference in scores of the three outcome variables, confirming the effectiveness of the intervention.

### Food selection

According to the Brunner-Munzel test, before the intervention, the selection of packaged foods between the control and intervention groups was not significantly different (*P* = 0.127), but it became significantly different after the intervention (*P* < 0.001). The pre-test and post-test differences were calculated, and the comparison of their median showed a significant difference between the two groups (*p* < 0.001), showing that the intervention group had a higher median (Table 2). This result means the intervention group chose healthier food.

**Table 2:** Comparison of the median scores of the choice of packaged foods in patients with acute coronary syndrome in the control and intervention groups before and after the intervention

The dependent variable	Intervention group		Control group		Brunner-Munzel Test	Relative effect size
	Median	Interquartile range	Median	Interquartile range		
**Before intervention**	16/5	16/25	19	24	*P* = 0/127	0.427
**Four weeks after the intervention**	50	8/25	18	24	*P* > 0/001^*^	0.918
**The difference in scores before and after**	26/5	22/5	0	0	*P* > 0/001^*^	0.957

^*^ *Statistically significant (p-Value < 0.05)*

### Food preference

The results of the Brunner-Munzel test shows that the food preferences before the intervention were not significantly different between the control and intervention groups (*P* = 0.852). But after the intervention, the intervention group obtained a higher score in terms of preference (*P* < 0.001). This was also seen in the comparison of the median of the pre-test and post-test differences (*p* < 0.001) (Table 3). This result means the intervention group preferred healthier food.

**Table 3:** Comparison of the median scores of packaged food preference in acute coronary syndrome patients in two control and intervention groups before and after the intervention

The dependent variable	Intervention group		Control group		Brunner-Munzel Test	Relative effect size
	Median	Interquartile range	Median	Interquartile range		
**Before intervention**	6	2	6	2	*P* = 0/852	0.491
**Four weeks after the intervention**	7	1	6	1/5	*P* < 0/001^*^	0.730
**The difference in scores before and after**	1	1	0	1	*P* = 0/001^*^	0.770

^*^ *Statistically significant (p-Value < 0.05)*

### Food consumption

The results of the Brunner-Munzel test shows that food consumption was not significantly different before the intervention between the two groups (*P* = 0.846), but the difference was significant after the intervention (*P* = 0.004). This was also seen in the comparison of the median of the pre-test and post-test differences (*p* = 0.011) (Table 4). This result means the intervention group consumed healthier food.

**Table 4:** Comparison of the median scores of packaged food consumption in acute coronary syndrome patients in the control and intervention groups before and after the intervention

The dependent variable	Intervention group		Control group		Brunner-Munzel Test	Relative effect size
	Median	Interquartile range	Median	Interquartile range		
**Before intervention**	124	16/5	125	24/5	*P* = 0/846	0.510
**Four weeks after the intervention**	138/5	18/25	131	18/5	*P* = 0/004^*^	0.640
**The difference in scores before and after**	13	14/75	5	16/5	*P* = 0/011^*^	0.624

^*^ *Statistically significant (p-Value < 0.05)*

## Discussion

This study found that the intervention group, who received routine patient education by hospital staff and TLL label training by the research team, scored higher than the control group, who received only routine patient education by hospital staff. This result means the intervention group chose, preferred, and consumed healthier food than the control group. These findings suggest that teaching the use of TLL can enhance the effectiveness of patient education and help reduce the burden of ACS in the long run.

The findings from this study are consistent with previous studies that reported positive effects of TLL on food purchasing and consumption behaviors [19, 20, 24]. However, they contrast with some studies that found no relationship between TLL and sales of healthier products [22, 23]. These discrepancies may be due to differences in the settings, populations, and methods of the studies.

To understand the complex nutritional information on food labels by the participants of the intervention group, even the participants who had a low level of education, we taught them to use the TLL label to prefer, choose and consume healthier food with only a few colors. Therefore, teaching the use of TLL can enhance the effectiveness of patient education and help reduce the burden of ACS in the long run.

For instance, Sonnenberg et al. conducted a study in an American hospital in 2010. They labeled foods sold in a cafeteria. A customer survey was carried out during a 2-week baseline period and a 7-week intervention period, with purchases monitored throughout. The study found that traffic light food labeling increased consumer awareness of food and beverage healthiness at the point of purchase. More consumers reported looking at nutrition information during the labeling intervention than the baseline period, and those who noticed the labels bought a higher proportion of healthy items.

Similarly, Olstad et al. used traffic light labels to improve food choices in a recreational and sports venue in Canada. They monitored product sales one week before and one week after food product labeling. The traffic light icons (green, yellow, and red) were placed on the menu screen or directly on the product shelf, and explanatory sheets were placed in the study environment. The study concluded that nutrition guidance labels effectively increased the sale of healthy foods and reduced the sale of unhealthy foods.

In Iran, Esfandiari et al. conducted a study to investigate the effectiveness of education on awareness, attitude, and performance based on the color indicator of appropriate food products. Students’ knowledge, attitude, and performance about the nutritional color indicator were measured using a questionnaire. Training was conducted face-to-face using an educational pamphlet. The questionnaire was filled out again by the participants 3–6 months after the training. The results show improvements in knowledge, attitude, and performance scores compared to before the training.

In contrast, Sacks et al.‘s study in the United Kingdom examined consumer food purchasing changes after TLL labeling to evaluate the label’s impact on the “healthiness” of purchased foods. The sales of two groups of products (prepared foods and sandwiches) were divided according to their healthiness. Comparisons were made before and four weeks after TLL labeling. The results show that the sales of prepared foods increased immediately after introducing the traffic light label. However, the sale of sandwiches did not change significantly after the introduction of labels. There was no association between changes in product sales and product healthiness [22].

A study conducted by Sacks and colleagues in Australia analyzed the changes in online food purchases over a period of 10 weeks, following the implementation of the Traffic Light Nutrition Information (TLNI) system. Four colored traffic light symbols indicated the amount of fat, saturated fat, sugar, and sodium. These were displayed for 53 products in the intervention store alongside the product list. No nutritional information was provided in the comparison shop. No association was seen between the sales changes in the two stores and the health value of the products [23].

This study had some limitations. First, it only assessed the short-term effect of patient education using TLL, so the long-term impact remains unknown. Second, it did not measure the effect of patient education using TLL on nutritional variables such as anthropometric, biochemical, clinical, and dietary indicators. Therefore, future studies should examine these endpoints and follow up with ACS patients for extended periods to evaluate the sustainability and impact of TLL label training on their health outcomes. The study also faced a limitation due to not completing the post-test, leading to the loss to follow-up of many participants. To mitigate this, we compared the demographic characteristics of participants who completed the post-test and those who didn’t. The analysis shows no significant differences in age, gender, or education level between these groups, suggesting that the attrition is unlikely to have introduced a significant bias in our results.

## Conclusion

The present study was a randomized controlled trial (RCT) aimed at assessing the efficacy of traffic light labeling (TLL) training on food selection, preference, and consumption among individuals who recently experienced acute coronary syndrome (ACS). The findings indicate that combining TLL training with standard hospital patient education yields superior results compared to the latter alone. Although the study was restricted to ACS patients, it is recommended that future research extend this to healthy individuals with cardiovascular risk factors, as well as those with a history of ACS who have been discharged for a significant period. The results underscore the importance of incorporating TLL education into nutritional guidance for ACS patients and suggest integrating TLL training into hospital education programs. Moreover, placing TLL educational posters in hospital cafeterias and near vending machines may foster healthier dietary habits among patients and staff. The study further proposes extending TLL training to other environments such as schools, workplaces, and supermarkets to promote healthier eating habits in the wider public. It is important to note, however, that this recommendation is only applicable to countries that use TLL on their products, and other countries may require different types of education. Additionally, this educational strategy should be considered as a single component of a patient’s education plan for healthy nutrition.

### Electronic supplementary material

Below is the link to the electronic supplementary material.

Supplementary Material 1

## Data Availability

The data that support the findings of this study are available from the corresponding author, [Reza Negarandeh], upon reasonable request.

## Abbreviations

TLL: Traffic light labeling
ACS: Acute coronary syndrome
