# Supplementary material for: The impact of healthy nutrition education based on traffic light labels on food selection, preference, and consumption in patients with acute coronary syndrome: a randomized clinical trial
Source: BMC Public Health. 2024 May 17;24:1332. doi: 10.1186/s12889-024-18805-2 (PMC11100130; doi:10.1186/s12889-024-18805-2)
Supplement: Supplementary file 1 — Supplementary Material 1 [file 12889_2024_18805_MOESM1_ESM.docx]

**Food selection questionnaire**

| **Questions** | Almost always | most of the time | sometimes | rarely | never |
| --- | --- | --- | --- | --- | --- |
| 1. **When I buy a food product for the first time, I read the nutritional label of the product.** |  |  |  |  |  |
| 1. **Nutritional information written on packaged food items...** | | | | | |
| A) It is enough |  |  |  |  |  |
| b) It is understandable. |  |  |  |  |  |
| c) It is reliable. |  |  |  |  |  |
| d) It is effective in my decision making and helps me choose healthier food. |  |  |  |  |  |
| 1. **Which of the following information are you specifically looking for when buying packaged food items?** | | | | | |
| A) Energy (calories) |  |  |  |  |  |
| b) Sugar |  |  |  |  |  |
| c) Fat |  |  |  |  |  |
| d) Salt |  |  |  |  |  |
| e) trans fatty acid |  |  |  |  |  |

**Food preference questionnaire**

**Which one do you usually prefer when buying packaged food items?**

| salty cheese □ | Low-salt cheese □ |
| --- | --- |
| Full fat milk □ | low fat milk □ |
| Soft drinks containing sugar □ | soft drinks without sugar □ |
| Contains hydrogenated oil (solid vegetable oil) □ | without hydrogenated oil (liquid oil) □ |

**Food consumption questionnaire**

**During the last four weeks, on average, how much of the following foods did you consume?**

| **Food product** | **Never** | **1-2 times** | **3 times** | **4-5 times** | **6 times or more** |
| --- | --- | --- | --- | --- | --- |
| Chocolate |  |  |  |  |  |
| Ice cream |  |  |  |  |  |
| Tea with sugar |  |  |  |  |  |
| Coffee with sugar |  |  |  |  |  |
| cokes |  |  |  |  |  |
| juice |  |  |  |  |  |
| Energy drink |  |  |  |  |  |
| ketchup |  |  |  |  |  |
| mayonnaise sauce |  |  |  |  |  |
| chocolate milk |  |  |  |  |  |
| Full-fat dairy |  |  |  |  |  |
| Processed Cheese |  |  |  |  |  |
| Sausage |  |  |  |  |  |
| Bologna |  |  |  |  |  |
| hamburger |  |  |  |  |  |
| Red Meat |  |  |  |  |  |
| Chicken |  |  |  |  |  |
| salty cheese |  |  |  |  |  |
| yogurt |  |  |  |  |  |
| pickled cucumber |  |  |  |  |  |
| Ready pickles |  |  |  |  |  |
| Canned foods |  |  |  |  |  |
| Butter |  |  |  |  |  |
| Pizza |  |  |  |  |  |
| fried food |  |  |  |  |  |
| chips |  |  |  |  |  |
| Sweet Cream |  |  |  |  |  |
| Nugget |  |  |  |  |  |
| Cakes and cookies |  |  |  |  |  |
| biscuit |  |  |  |  |  |
| Donut |  |  |  |  |  |
| Solid vegetable oil |  |  |  |  |  |
